# Direct Measurement of Perchlorate Exposure Biomarkers in a Highly Exposed Population: A Pilot Study

**DOI:** 10.1371/journal.pone.0017015

**Published:** 2011-03-04

**Authors:** Paul English, Ben Blount, Michelle Wong, Lori Copan, Luis Olmedo, Sharyle Patton, Robert Haas, Ryan Atencio, Juhua Xu, Liza Valentin-Blasini

**Affiliations:** 1 California Environmental Health Tracking Program, California Department of Public Health, Richmond, California, United States of America; 2 Division of Laboratory Sciences, Centers for Disease Control and Prevention, Atlanta, Georgia, United States of America; 3 Environmental Health Investigations Branch, California Department of Public Health, Richmond, California, United States of America; 4 Comité Cívico del Valle, Brawley, California, United States of America; 5 Commonweal, Bolinas, California, United States of America; 6 Food and Drug Laboratory Branch, California Department of Public Health, Richmond, California, United States of America; 7 California Department of Toxic Substances and Control, El Centro, California, United States of America; Brigham & Women's Hospital, and Harvard Medical School, United States of America

## Abstract

Exposure to perchlorate is ubiquitous in the United States and has been found to be widespread in food and drinking water. People living in the lower Colorado River region may have perchlorate exposure because of perchlorate in ground water and locally-grown produce. Relatively high doses of perchlorate can inhibit iodine uptake and impair thyroid function, and thus could impair neurological development in utero. We examined human exposures to perchlorate in the Imperial Valley among individuals consuming locally grown produce and compared perchlorate exposure doses to state and federal reference doses. We collected 24-hour urine specimen from a convenience sample of 31 individuals and measured urinary excretion rates of perchlorate, thiocyanate, nitrate, and iodide. In addition, drinking water and local produce were also sampled for perchlorate. All but two of the water samples tested negative for perchlorate. Perchlorate levels in 79 produce samples ranged from non-detect to 1816 ppb. Estimated perchlorate doses ranged from 0.02 to 0.51 µg/kg of body weight/day. Perchlorate dose increased with the number of servings of dairy products consumed and with estimated perchlorate levels in produce consumed. The geometric mean perchlorate dose was 70% higher than for the NHANES reference population. Our sample of 31 Imperial Valley residents had higher perchlorate dose levels compared with national reference ranges. Although none of our exposure estimates exceeded the U. S. EPA reference dose, three participants exceeded the acceptable daily dose as defined by bench mark dose methods used by the California Office of Environmental Health Hazard Assessment.

## Introduction

Perchlorate occurs in the environment from both natural and man-made sources. It is primarily synthesized for use as an oxidant in solid rocket propellant. Perchlorate has been detected in food and drinking water from various regions of the U.S. [1]–[3], and human exposure to perchlorate is widespread in the U.S. population [4]. At high doses (mg/kg of body weight/day), perchlorate can affect the ability of the thyroid to absorb iodine and can limit the production of thyroid hormones, which are important for proper development in children [5]. Continued inhibition of iodine uptake can lead to hypothyroidism, which can result in metabolic problems in adults and abnormal development during gestation and infancy. Low doses (µg/kg/day) of perchlorate have been associated with decreased thyroxine and increased thyroid-stimulating hormone levels in women with low urinary iodine levels [6]. Even small changes in thyroid hormone levels are cause for concern, as mild hypothyroidism during pregnancy has been associated with subtle cognitive defects in children [7]–[9]. Other compounds that inhibit iodine uptake are thiocyanate (SCN) and nitrate (NO_3_) [10]. These compounds are also present in dietary and water sources, and SCN is a major metabolite of cyanide found in cigarette smoke, so these compounds are important to consider when examining perchlorate's antithyroid effects [11].

**Figure 1:**
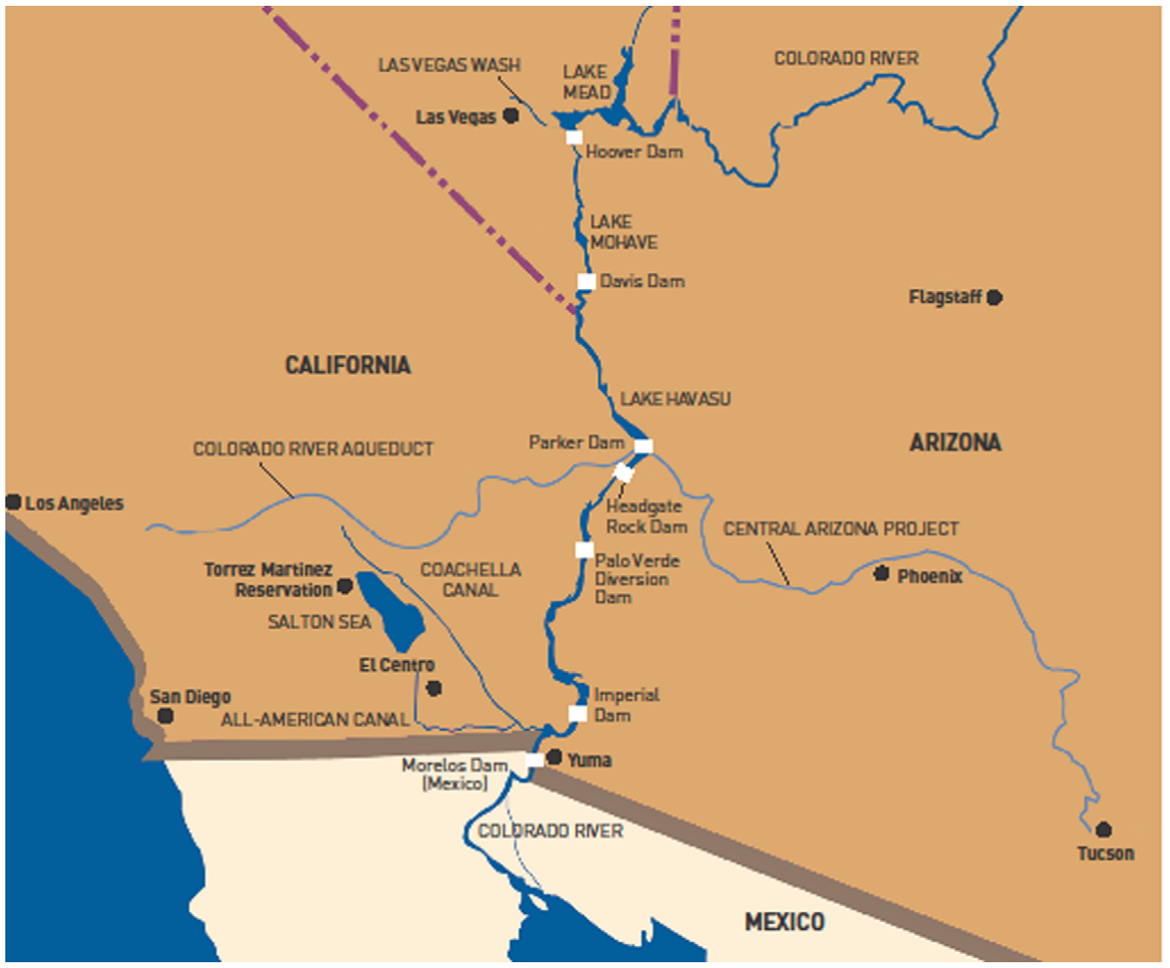
Map showing the Lower Colorado River from the source of perchlorate contamination in the Las Vegas Wash to the All-American Canal in the Imperial Valley. Source: U.S. Bureau of Reclamation.

Although perchlorate exposure is widespread in the U.S. population, some locations may have higher exposure than others. One such location is the Lower Colorado River region. In Nevada, ammonium perchlorate manufacturing activities contaminated ground and surface waters, and eventually, Lake Mead and the Colorado River (Figure 1). The U.S. Environmental Protection Agency (EPA) and the State of Nevada are currently overseeing cleanup operations for the area. The Colorado River is a primary source of drinking water for 15 million–20 million people in Arizona, Nevada, and California and also serves as the sole source of irrigation water for California's Imperial Valley. Imperial County has approximately 160,000 residents, and about 20% of families are below the poverty level. Potentially elevated chemical exposures in this low-income population may raise issues of environmental equity.

The California Environmental Health Tracking Program (CEHTP) conducts surveillance on environmental exposures and environmentally-related chronic diseases. Due to concerns expressed about levels of perchlorate in produce grown in the lower Colorado River region [12] and the lack of human exposure studies [13], CEHTP partnered with the Centers for Disease Control and Prevention (CDC), the California Department of Public Health (CDPH) Food and Drug Laboratory Branch (FDLB), the California Department of Toxic Substances Control (DTSC), and 2 nongovernmental organizations (Commonweal and Comité Cívico del Valle) to measure perchlorate exposure in the Imperial Valley among persons consuming locally grown produce.

## Materials and Methods

We recruited a convenience sample of 31 residents living in Imperial County, California, from attendees of a community meeting related to local environmental concerns. Written informed consent was obtained from each participant. Adult residents who consumed local produce were eligible to participate. We designed a short questionnaire to elicit information on participants' demographic and personal characteristics (e.g., gender, age, weight, and height), smoking history, primary source of drinking water, water filter use in the home, water consumption, and source of produce. We also asked participants to complete a 24-hr dietary recall and a food diary to be completed for the 24-hr study period. All materials were available in Spanish and English. Written informed consent was obtained from all participants. All human subject protocols were approved by the Centers for Disease Control and Prevention's institutional review board. Participants received their individual results from the laboratory tests of urine, produce, and water and were also provided materials to help interpret the results. Group findings were presented at a participant meeting, and a local clinician was available for consultation with participants for any health concerns related to the study.

In addition, participants were asked to collect samples of all drinking water and all local produce consumed during a 24-hr period. Study staff provided standardized containers to participants for collection of all samples. Participants were instructed to collect approximately 2 ounces of each produce sample in a plastic bag. Produce samples were stored in participants' freezers until collected by study staff. Participants were also instructed to collect a 24-hr urine specimen, beginning after the last void of the day. Urine was chilled in coolers or participants' refrigerators during sample collection and transported in coolers by study staff. Total urine volume was measured volumetrically and recorded for each study participant. Urine sample aliquots (5 mL) were removed from the vessels storing the 24-hr urine samples, transferred to Cryovials®, and stored at −20°C at the Imperial County Public Health Laboratory until shipped to CDC on dry ice. Produce samples were shipped to the CDPH FDLB, and water samples were analyzed by the DTSC.

Water samples were analyzed to detect perchlorate by using EPA method 6850 (liquid chromatography-mass spectrometry) with a detection limit of 1 µg/L. Perchlorate was resolved by high-performance liquid chromatography from the sample matrix, ionized by using negative electrospray ionization, and partially fragmented by mass spectrometry (MS). Perchlorate was further fragmented into daughter ions upon collision with an inert gas and detected by tandem MS using mass-to-charge (m/z) ratios 83 (-ClO_3_), 85(-^37^ClO_3_) and 89(-Cl^18^O_3_) [14]. Quantitation and identification of perchlorate were performed by comparing the ratios of the primary perchlorate mass transitions to internal standard mass transitions and by using retention times. Upon positive detection, 2 samples were further analyzed to confirm accuracy and precision. Samples were transferred to a clean centrifuge tube and evaporated to dryness under a stream of nitrogen at 70°C. The samples were then resuspended in 1.0 mL of deionized water.

Produce was analyzed for perchlorate by using ion chromatography-tandem MS [15]. The produce was chopped with a food processor (Robot Coupe USA, Inc., Jackson, MS, USA) until the matrix was homogeneous. A portion of chopped produce (10 g) was transferred to a 50 mL polypropylene tube, and 100 µL of 3.0 µg/mL (300 ng) labeled internal standard was added. This material was acidified by addition of 20 mL of 1% (vol/vol) sodium hydroxide acetic acid, hand-shaken for 2 minutes and centrifuged at 2000 rpm for 15 minutes. Supernatant (5 mL) was passed through a 500 mg–6 mL Supelclean™ ENVI-Carb™ cartridge (Sigma-Aldrich, Milwaukee, WI, USA) preconditioned with 6 mL of water by applying vacuum such that the flow rate was approximately 100 µL/second. This extract was filtered through a 0.20 µm PTFE filter and analyzed by ion chromatography-tandem MS using a Waters Quattro micro™ API triple-quad mass spectrometer operating in negative ion multiple reaction monitoring mode. The m/z transition from 99 to 83 was primary for quantitating perchlorate, and the m/z transition of 101 to 85 was confirmatory. ^18^O_4_-labeled sodium perchlorate (ICON Services, Inc., Summit, NJ, USA) served as internal standard for monitoring the m/z transitions from 107 to 89 and 109 to 91 for quantitation and confirmation, respectively. The limit of quantitation was 1 ng/g. All produce results were reported using the wet weight of the edible portion of produce.

Perchlorate and related anions (thiocyanate, nitrate, and iodide) were measured in urine by ion chromatography-tandem MS [16]. Analytes were quantified on the basis of the peak area ratio of mass transitions for analyte to stable isotope-labeled internal standard. Method detection limits were 0.05 µg/L, 0.50 µg/L, 10 µg/L, and 500 µg/L, for perchlorate, iodide, thiocyanate, and nitrate, respectively. Analytic accuracy and precision were tested by concurrent analysis of quality control materials in the same analysis batch as the unknown specimen. Reported results met the accuracy and precision specifications of the quality control and quality assurance program of the Division of Laboratory Sciences, National Center for Environmental Health, CDC [16]. All analytes were detected in all samples with the exception of 4 urine samples that did not contain measurable levels of nitrate; nitrate levels in these two samples were assigned an imputed value of half the detection limit for calculating the geometric mean of the distribution.

Urinary creatinine concentrations were determined by using an automated colorimetric method on a Roche/Hitachi Modular Analytics SWA system (Roche Diagnostics Corp., IN, USA). Creatinine excretion rate (g/24-hr) was calculated by multiplying measured urinary creatinine concentration by the total volume of 24-hr urine collected. The calculated 24-hr creatinine excretion rate was then compared with expected 24-hr creatinine excretion rate based on the study participant age, sex, height, and weight [17], [18], according to a formula in which k = 1.93 for males and 1.64 for females:
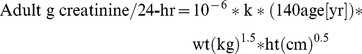
We computed perchlorate dose in urine by multiplying the perchlorate concentration by the 24-hr urine volume and dividing the product by body weight (µg/kg/day). Total iodine intake was estimated by multiplying urine iodide concentration by 24-hr urine volume.

Final perchlorate doses were compared to the U.S. EPA Reference Dose of 0.70 µg/kg/day [19]. We also compared measured perchlorate doses with an “acceptable daily dose” computed based on the current methodology of the California Office of Environmental Health Hazard Assessment (OEHHA) (C. Steinmaus, personal communication). In its Public Health Goal for Perchlorate, the OEHHA computed the health-protective water concentration for perchlorate [20] as follows:
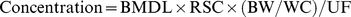
In this formula, BMDL = the lower limit of a one-sided 95% CI of a perchlorate dose that reduces mean thyroidal iodine uptake by 5%; RSC = relative source contribution; BW/WC = the ratio of body weight to tap water consumption rate; and UF = an uncertainty factor of 10 to account for sensitive populations such as pregnant women and infants. Using current OEHHA methodology, we computed an acceptable daily dose (ADD) as follows:
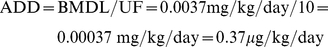
Survey questionnaire data were double-entered into Excel speadsheets (Excel version 2003, SP3, Microsoft, Redmond, WA, USA) and then imported into SAS for analysis (SAS version 9.1.3., SAS Institute, Inc., Cary, NC, USA). We calculated geometric means and CIs for perchlorate, nitrate, thiocyanate, and total iodine intake. For each person, perchlorate concentration was summed for all produce sampled. Total number of servings of dairy products (i.e., milk, yogurt, and ice cream) was also summed for each person. We used the nonparametric Kruskal-Wallis test to compare levels of perchlorate dose by total dairy servings and perchlorate concentration in produce [21]. The perchlorate dose was log-normally distributed, so total servings of dairy and total concentration of perchlorate in produce were regressed on the log-normal perchlorate dose in linear regression models.

## Results

### Participant Characteristics

The age of participants ranged from 18 to 88, with an average of 41.1 years (Table 1). Among participants, 22.6% had no high school education, 12.9% were high school graduates, and 12.9% had at least a college degree. Almost all (97%) participants were Hispanic, except for 1 non-Hispanic white. Eighty-one percent of participants elected to have the interview conducted in Spanish. Sixty-five percent of the participants were female, none of whom reported being pregnant at the time of the interview. Seventy-five percent of the women were of reproductive age (ages 15–49). Only 1 participant reported being a tobacco smoker. Two respondents reported working in agriculture. When asked to identify the primary source of drinking water in the home, 36% of participants reported bottled water, 23% reported tap water, and the remaining respondents reported various sources, such as well water, delivery truck, and grocery store dispensers. Forty-five percent of participants consumed <25% of their water away from home. Total dairy consumption averaged 1.05 servings per person per day, with a range of 0–5 servings.

**Table 1:** Participant Characteristics, Imperial County, 2009.

	N	%
Total Participants	31	100
Age		
18–25	6	19.4
26–35	7	22.6
36–45	7	22.6
46–55	5	16.1
>55	6	19.4
Gender		
Female	20	64.5
Male	11	35.5
Education		
No high school	7	22.6
Some high school	2	6.5
High school graduate	4	12.9
Some college	14	45.2
College Graduate	1	3.2
Graduate		
or Professional Degree	3	9.7
Ethnicity		
Hispanic	30	97
Non-Hispanic	1	3
Primary source of drinking water at home		
Bottled water	11	35.5
Tap water (filtered)	5	16.1
Tap water (unfiltered)	2	6.5
Well water (filtered)	1	3.2
Other	12	38.7
Amount of water consumed away from home		
<25%	14	45.2
25–50%	12	38.7
51–75%	4	12.9
75–99%	1	3.2

### Water and Produce Perchlorate Results

Results of water confirmation analyses showed consistency with original, primary sample results. Additionally, method blanks, method standard recoveries, and sample duplicate analyses' results were all within accepted control limits and percentages. Only 2 of 68 water samples had detectable levels of perchlorate (2.4 and 2.5 µg/L perchlorate), both of which were less than half the California regulatory standard of 6 µg/L. Perchlorate levels in the 79 produce samples ranged from nondetectable to 1816 ppb (ng of perchlorate per g of net weight of edible portion) (Table 2). The highest perchlorate levels were detected in nopales (cactus) (maximum of 1398 ppb) and quelites (Mexican greens) (2 samples tested, with levels of 1720 and 1816 ppb, respectively). Many produce categories, such as lettuce, broccoli, grapefruit, tomatoes, watermelon, and corn, had perchlorate levels ≤4 ppb. For 14 samples with perchlorate levels falling below the detection limit, we used an imputed value of half the detection limit. The mean perchlorate level of all produce was 80.8 ppb. Most produce types had too few samples per type to compute a representative average. Among those persons who provided produce (n = 24), the mean perchlorate level in their produce was 266 ppb per person. Produce perchlorate levels were in the range of similar kinds of produce tested by the U.S. Food and Drug Administration (FDA) in nationally representative samples, except for celery and cucumbers, which were higher in our samples, and tomatoes and watermelon, which were lower (Table 2).

**Table 2:** Perchlorate levels found in locally grown produce compared to FDA survey levels,^a^ Imperial Valley, CA, 2009.

Produce Type	Number of samples	Perchlorate range (ppb)	FDA data*(ppb)
Broccoli	2	2.1–3.5	1.3–8.3
Canary melon	1	3.8	NA^b^
Cantaloupe	10	1.9–7.9	1.4–70.3
Carrot	3	3.9–8.4	Non detect – 7.7
Celery	2	22.9–28.0	Non detect – 2.2
Corn	3	ND-3.6	Non detect
Corn & squash	2	2.2–4.1	NA
Cucumber	2	171.4–439.8	Non detect – 64
Grapefruit	2	2.3–2.7	Non detect
Green pepper	1	33.9	5.4–26.7
Lettuce	2	<1	Non detect – 6.7
Lemon	1	1.4	NA
Melon (non specified)	4	1.6–6.4	NA
Nopal (cactus)	5	37.9–1398.4	NA
Onion (red)	2	<1–293.7	NA
Onion (non specified)	5	<1–3.8	Non detect
Onion (white)	1	1	NA
Orange	1	5	Non detect – 5.4
Potato	4	<1–2.0	Non detect – 1.0
Quelites (Mexican greens)	2	1719.9–1816	NA
Tomato	7	<1–3.3	54.1–102
Watermelon	17	<1–3.7	Non detect – 42.6
Total Samples	79		

### Urine Results

The 24-hr creatinine output was normally distributed with a mean of 1.33 g/day (SD = 0.494 g/day). The 24-hr creatinine output was compared with the predicted 24-hr creatinine output [17] to evaluate completeness of urine collection. The average ratio of measured-to-predicted 24-hr creatinine was 1.03, indicating good compliance of the study participants for collecting all urine within the defined 24-hr study period.

Perchlorate concentrations in urine ranged from1.08 µg/L to 32.2 µg/L, with a geometric mean of 6.44 µg/L (Table 3). The 24-hr perchlorate dose in urine ranged from 0.02 to 0.51 µg/kg of body weight/day. The geometric mean perchlorate dose for study participants was 0.11 µg/kg/day (95% CI, 0.08–0.15). Perchlorate concentrations and doses were higher in the Imperial Valley study participants compared to reference values from the National Health and Nutrition Examination Survey (NHANES).

**Table 3:** Range and Geometric Means of Major Analytes, Imperial County, 2009, compared to NHANES^a^.

Analyte	Min	Max	GM	95% C.I.	NHANES GM(95% CI)
Perchlorate (µg/L)	1.08	32.2	6.44	4.83–8.58	3.35 (3.08–3.65)
Perchlorate					
(µg/g creatinine)	1.24	37.4	6.98	5.10–9.57	3.46 (3.20–3.73)
Perchlorate dose					
(µg/kg/day)	0.02	0.51	0.112	0.082–0.152	0.066 (0.060–0.071)
Thiocyanate					
(µg/g creatinine)	178	2715	816	634–1051	1500 (1400–1620)
Nitrate					
(µg/g creatinine)	<700	152,110	21,770	11,595–40,876	44,500 (42,300–46,800)
Total iodine intake					
(µg/day)	40.2	679	158	121–206	N/A

a Representative data for adult U. S. residents from the National Health and Nutrition Examination Survey, 2001–2002 (Blount et al. 2006; Blount et al. 2007).

The geometric means for urine levels of thiocyanate and nitrate were 816 and 21,800 µg/g creatinine, respectively, compared to geometric means for the general U. S. population of 1500 and 44,500 µg/g creatinine based on data from NHANES 2001–2002 (Blount, unpublished data). The geometric mean for daily iodine intake was 158 µg/day, which is slightly higher than the Recommended Daily Intake (RDI, 150 µg/day) established by the U.S. FDA . Eighteen participants, including 10 of the 15 women of reproductive age, had daily iodine intakes below 150 µg/day.

**Figure 2:**
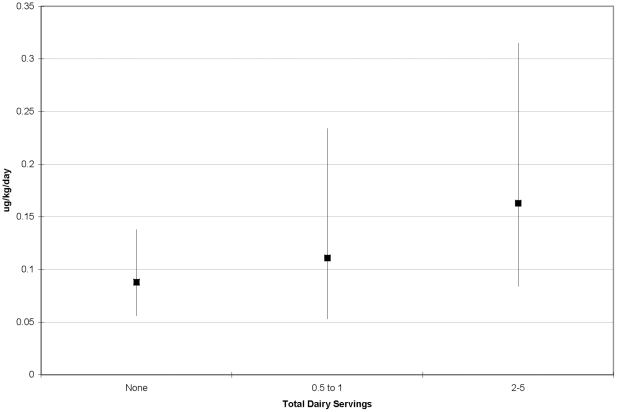
Geometric mean perchlorate dose in urine (µg/kg of body weight/day) and 95% confidence intervals in study participants by total dairy servings, Imperial County, California, 2009.

**Figure 3:**
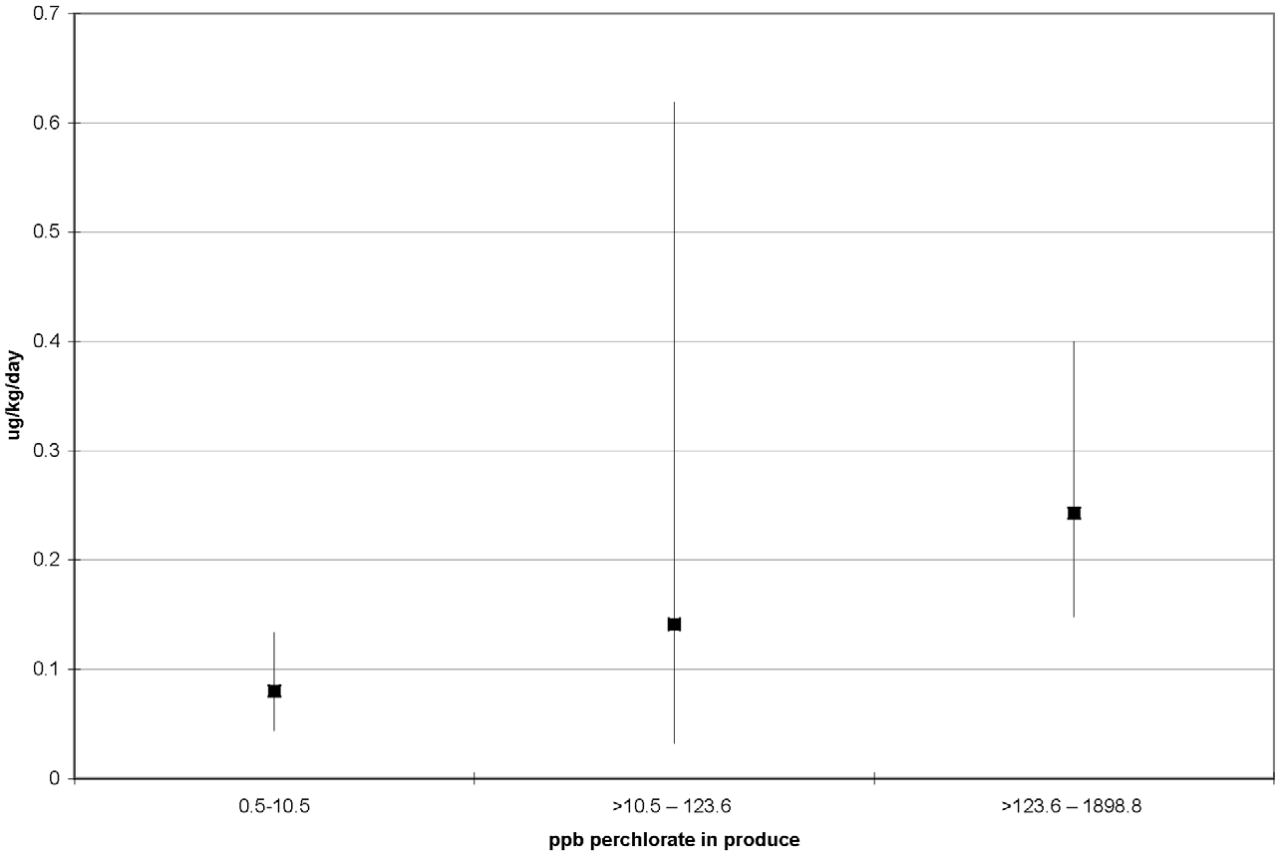
Geometric mean perchlorate dose in urine (µg/kg of body weight/day) and 95% confidence intervals in study participants by perchlorate concentration in produce for each participant, Imperial County, California, 2009.

When stratifying perchlorate dose by total dairy servings, geometric means of perchlorate dose increased with increasing dairy consumption (Figure 2) (nonparametric Kruskal-Wallis test, p = 0.24). In a linear regression model on log perchlorate dose, each serving of dairy products was associated with a 24% increase in urine perchlorate levels (p = 0.04; r^2^ = 0.14). Perchlorate dose in urine showed a similar increase with categories of perchlorate concentration (using natural breaks in perchlorate distribution) in consumed produce (Figure 3) (nonparametric Kruskal-Wallis test, p = 0.03). Each 10 ppb of perchlorate in produce was associated with a 1% increase in log perchlorate dose (p = 0.09; r^2^ = 0.13).

## Discussion

In this study of perchlorate exposure among Imperial County residents, we found perchlorate exposure doses that were, on average, 70% higher than the NHANES U.S. reference population (Table 3). Although all study participants had measurable perchlorate levels in their urine, none of the exposure doses exceeded the U.S. EPA reference dose of 0.70 µg/kg/day. We also compared perchlorate exposure estimates with the acceptable daily dose (0.37 µg/kg/day) derived by using current methodology of the California Office of Environmental Health Hazard Assessment. Three study participants had perchlorate exposure dose estimates in excess of this acceptable daily dose. The health implications of this perchlorate exposure are unknown.

The developing fetus is thought to be a particularly sensitive life stage for perchlorate exposure [5]. Measurement of perchlorate in amniotic fluid confirms that perchlorate can cross the placenta [22]. Therefore, we further examined perchlorate exposure in the 48% of our study population who were women of reproductive age. We found that 1 of these women exceeded the acceptable daily dose calculated by using OEHHA methodology. Among the 15 women of reproductive age in our study, 10 (75%) had low iodine intake (below the RDI). Continued perchlorate exposure above the OEHHA threshold among these women could increase their risk of subclinical hypothyroidism, which has been linked to subtle cognitive defects in children. To what degree perchlorate exposure contributes to the prevalence of subclinical hypothyroidism (2%–3%) in women of reproductive age is unknown [23].

Perchlorate exposure is of health concern because perchlorate inhibits uptake of iodine, which is essential for thyroid function. Thiocyanate (elevated after exposure to cyanide in tobacco smoke, for example) and nitrate may interact with perchlorate in an additive fashion that also inhibits iodine uptake [10]. The levels of thiocyanate and nitrate found in this study were comparable to or lower than levels found previously in other populations [4]. We also evaluated urinary levels of iodine in this population. The geometric mean for iodine was found to be 158 µg/day, indicating a generally adequate intake of iodine. If a person's estimated iodine intake was less than the recommended daily intake of 150 µg/day, then he or she was advised to increase iodine intake through diet or by taking iodine-containing supplements. Additionally, for study participants who chose to add salt to their food, we mentioned iodized salt in small amounts as an effective iodine source.

We did not find that the drinking water samples collected in the 24-hr study period had significant levels of perchlorate. Only 2 samples had detectable levels (>1 µg/L detection limit), and both were below the Maximum Contaminant Limit (6 µg/L) for California. Conversely, we found several samples of produce consumed by participants to have high levels of perchlorate, notably nopales (opuntia cactus) and quelites, with levels exceeding 1700 ppb. These results are consistent with other published measurements of relatively high levels of perchlorate in opuntia cactus [24]. Perchlorate levels in some produce tested, such as cantaloupe, grapefruit, pepper, broccoli, and lemon, are in the same range as levels that have been reported previously [1], [27], and others, including watermelon and tomato, had lower levels than previously detected. We found moderate perchlorate contamination in cucumbers (439.8 ppb), similar to previously published results [25].

Consistent with the scientific literature, we found that perchlorate dose was related to intake of dairy products and produce. Dairy products, fruits and vegetables have been characterized by other scientists as contributing significantly to perchlorate intakes [26], [27]. In our study we found that each 10 ppb of perchlorate measured in produce was related to a marginally-significant 1% increase in estimated perchlorate dose (p = 0.09). Similarly we found that each serving of milk/dairy products, based on questionnaire data, was associated with a 24% increase in estimated perchlorate dose (p = 0.04). While our regression modeling indicates that dairy consumption and produce perchlorate levels are directly related to estimated perchlorate dose, these models only explained a relatively small amount of the total variance in estimated perchlorate dose in the study population (13–14%). The most plausible explanation of the low relatively R^2^ of our regression models is that we could not include a variable for the total intake amount of perchlorate from produce (no serving size data) and milk/dairy products (no measurement of perchlorate levels of serving size). Furthermore, the physiological half life of perchlorate in the human body (∼8 hrs) would lead to imperfect overlap between questionnaire data and perchlorate excreted into the 24-hr urine, and consumption of other food items besides local produce during the study period would introduce further variability.

This study had several strengths and limitations. To our knowledge, this is the first effort to directly measure perchlorate exposure biomarkers in this population with potentially elevated exposure. In addition to perchlorate, we also measured toxicologically-related anions thiocyanate, nitrate, and iodide in each study participant's urine. Our exposure assessment was further strengthened by measuring these anions in a 24-hr urine sample so that the data was less variable than spot urine measurements. Furthermore, this biomonitoring data was paired with simultaneously collected produce and drinking water samples to assist in identifying potential exposure sources. However, we were able to only assess dairy consumption through interview and were unable to directly measure perchlorate levels in dairy samples. Instead, we reference previously published perchlorate levels in dairy milk in this region [12].

Our perchlorate dose estimate method assumed that exactly 24 hrs worth of urine was collected. Comparison of measured 24-hr creatinine with predicted 24-hr creatinine indicates that, on average, the predicted and measured creatinine excretion agree remarkably well (average ratio = 1.03). However, 1 woman excreted only 54% of the creatinine expected for her age and body weight, perhaps due to differences in lean body mass or to incomplete collection of the urine samples within the 24-hr period. Thus, our exposure estimates for this 1 study participant may be biased towards being lower than actual exposure levels.

Additional limitations include the small convenience sample of study participants which prevent us from generalizing these results to Imperial County residents and the single sampling day, which did not enable us to study variability of exposure across days and seasons. Additionally, we did not examine any health or thyroid endpoints.

In conclusion, we found that our sample of 31 Imperial Valley residents had higher perchlorate dose levels compared with national reference ranges. Additionally, 3 participants exceeded the acceptable daily dose calculated by using methods of the California Office of Environmental Health Hazard Assessment. Several produce samples collected had high perchlorate levels, exceeding 1700 ppb. Continued biomonitoring of perchlorate exposure in this population could help evaluate whether reducing perchlorate contamination of the Colorado River over time leads to reduced human exposure.
